# Effect of HBsAg expression in liver tissue on prognosis of hepatocellular carcinoma after minimally invasive interventional therapy

**DOI:** 10.3389/fonc.2023.1106333

**Published:** 2023-03-09

**Authors:** Biyu Liu, Qi Wang, Tingting Mei, Jiasheng Zheng, Wenfeng Gao, Chunwang Yuan, Kang Li, Yonghong Zhang

**Affiliations:** ^1^ Research Center For Biomedical Resources, Beijing You’an Hospital, Capital Medical University, Beijing, China; ^2^ Interventional Therapy Center For Oncology, Beijing You’an Hospital, Capital Medical University, Beijing, China

**Keywords:** prognosis, HBsAg, interventional therapy, hepatocellular carcinoma, pathology

## Abstract

**Background:**

The aim of this study was to investigate the association between pathologic markers and prognosis in patients with hepatocellular carcinoma who received transcatheter chemoembolization combined with locoregional ablation therapy.

**Methods:**

This retrospective study included 111 hepatitis B virus (HBV)-associated hepatocellular carcinoma (HCC). All patients underwent transcatheter arterial chemoembolization (TACE) combined with locoregional ablation therapy, and received core needle biopsy before therapy in Beijing You ‘an Hospital affiliated to Capital Medical University from January 1, 2013 to December 31, 2016. Demographic, pathological indicators and clinical laboratory data were collected. The cumulative recurrence-free survival (RFS) and overall survival (OS) were calculated and compared by Kaplan-Meier method and Log-rank test, and Cox proportional risk model was used to screen for independent predictors of recurrence and long-term prognosis in HCC patients.

**Results:**

There was a correlation between HBsAg expression in liver tissue and prognosis of HCC patients. Patients with negative HBsAg expression had longer 1-,3- and 5-year RFS rates than positive HBsAg expression (78.3%, 43.5%, 30.4% and 58.5%, 24.5%, 17.0%, P=0.018). Meanwhile,the postoperative 1-,3-and 5-year OS rates of HCC patients in the negative HBsAg expression group were significantly higher than those of HCC patients in the positive HBsAg expression group (100%, 89.1%, 80.4% and 100%, 75.5%, 58.5%, P=0.008).

**Conclusions:**

The prognosis of patients with hepatocellular carcinoma with negative HBsAg expression was better than that with positive HBsAg expression. Accordingly, the expression of the liver HBsAg before combined therapy was a prognostic indicator for OS and RFS. For patients with liver HBsAg positive, follow-up should be strengthened and corresponding intervention measures should be taken to improve prognosis.

## 1 Introduction

There were 910,000 new cases and 830,000 deaths of hepatocellular carcinoma (HCC) worldwide, it was the sixth most common cancer globally and the third leading cause of cancer-related mortality. In China, HCC has 410,000 new cases and 390,000 deaths, ranking fifth and second in morbidity and mortality and HCC has become a health problem in China that cannot be ignored and has increased the medical burden (1).. HCC is usually developing in the context of chronic liver disease, which is mainly associated with hepatitis B virus (HBV) or hepatitis C virus (HCV) infection, alcohol intake or the metabolic syndrome. However, due to the low early diagnosis rate and high postoperative recurrence rate of HCC, the long-term prognosis of liver cancer is poor, and the 5-year survival rate is only about 12.1% (2, 3).

Due to the high sensitivity and specificity of computerized tomography(CT) or magnetic resonance imaging(MRI)for HCC detection, the diagnosis of liver cancer in most cases does not depend on pathological examination (4, 5). However, the microenvironment of liver is closely related to the occurrence and development of HCC, as it allows for a definitive diagnosis and provides prognostic information of patients (3). Ablation has similar efficacy to surgical for patients with early-stage HCC (6); and transarterial Chemoembolization (TACE) combined with ablation can effectively reduce the local blood supply of tumors and downstage tumors, and has unique advantages in preventing postoperative bleeding. TACE combined with ablation can make up for the disadvantages of TACE or ablation alone (7).

Liver microenvironment is of great significance for the occurrence and development of hepatocellular carcinoma. Therefore, this study aimed to investigate the predictive value of pathological indicators of HCC patients who received combined therapy.

## 2 Patients and materials

### 2.1 Study subjects

**Figure 1:**
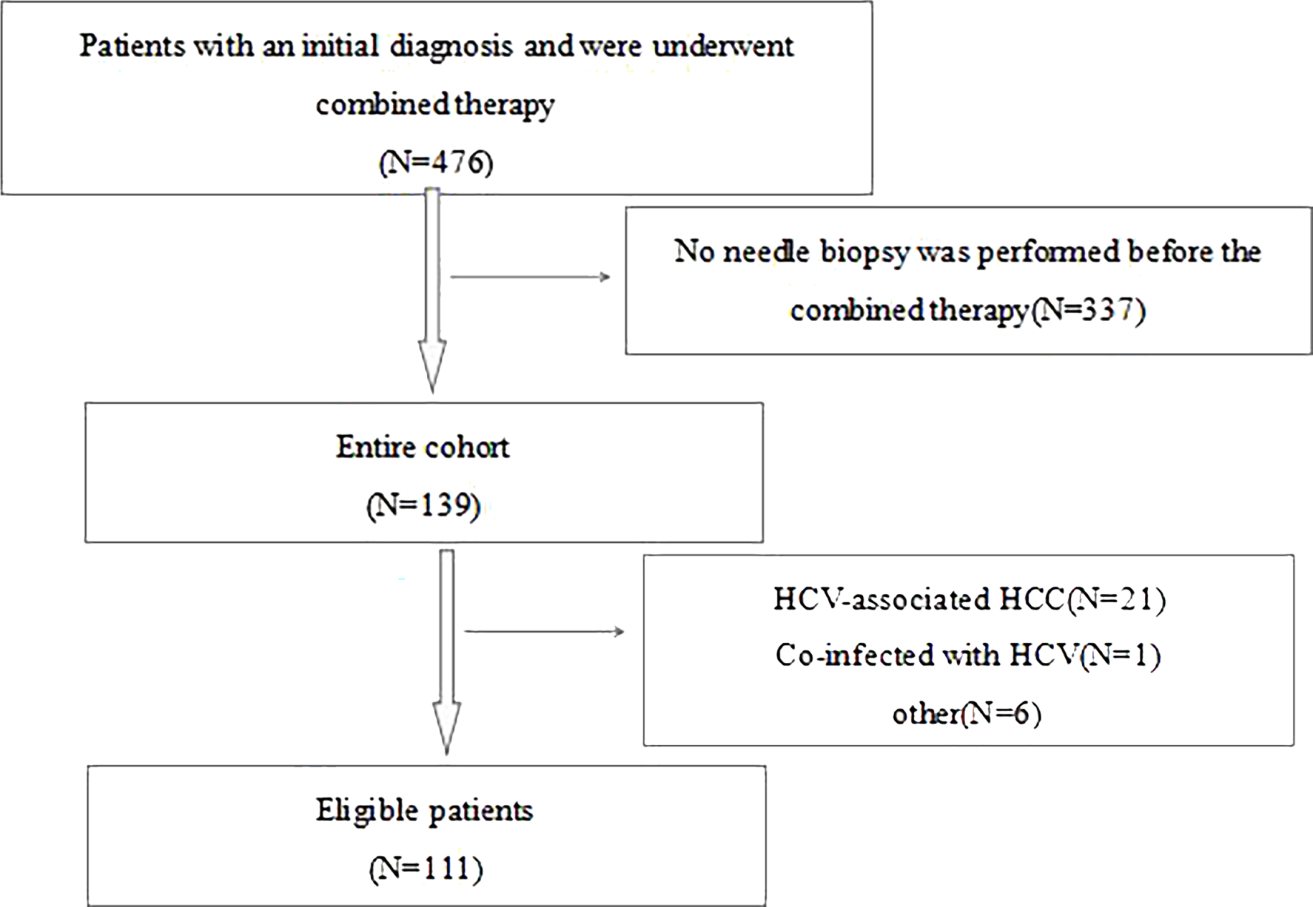
Flowchart of study participants. HCC, hepatocellular carcinoma; hepatitis B virus; HCV, hepatitis C virus.

This is a retrospective study of 111 patients with HCC who received combination therapy from January 1, 2013 to December 31, 2016 at Beijing Youan Hospital (Beijing, China). Most of the patients were early-stage small HCC and all initial treatment patients. For the subsequent diagnosis and treatment plan and prognosis, all patients voluntarily underwent needle biopsy. Biopsy is generally performed under the guidance of ultrasound. Contraindications for percutaneous liver biopsy mainly include patients with bleeding tendency, patients with severe cardiopulmonary disease, massive ascites, severe extrahepatic obstructive jaundice, lack of consciousness, inability to cooperate, suspected hemangioma or other vascular tumors, and suspected echinococcus cyst in the liver. All patients participating in the study were required to meet the following inclusion criteria: 1) age between 18 and 75 years old; 2) the combination of TACE plus ablation is the primary treatment method; 3) Child-Pugh class A or B; 4) no other malignancies that may affect prognosis; 5) all patients underwent core needle biopsy before combined therapy; and 6) HBV-associated HCC. The exclusion criteria were described below: 1) imaging evidence of invasion of the main branches of the portal/hepatic veins; 2) presence of extrahepatic metastases; 3) severe coagulation disorders; 4) incomplete ablation; 5) secondary liver cancer; 6) co-infection with HCV; and 7) missed follow-up examinations **Figure 1**.

Demographic information, pathological indicators and clinical laboratory data were collected: 1) demographic and etiology indicators, such as age, sex, history of hypertension and somking, serum HBsAg; 2) tumor-related indices, such as the number and size of tumors, and alpha-fetoprotein (AFP) level; 3) liver function indices, including cirrhosis, alanine aminotransferase (ALT), aspartate aminotransferase (AST), total serum bilirubin (TBIL), serum albumin, globulin, and γ-glutamyl transpeptidase(γ-GT); 4) routine blood examinations, such as neutrophil count (NEU), lymphocyte count (LYM), platelet count (PLT); 5) blood coagulation indicators: including prothrombin time (PT), prothrombin activity (PTA), fibrinogenic (Fib); and 6) pathological indicators: Ki67, P53, HBsAg, Glypican-3 (GPC3), the degree of tumor differentiation. The study was conducted in accordance with the 1964 Declaration of Helsinki and approved by the Ethics Committee of You’an Hospital in Beijing. Information on these patients was kept confidential. As a minimal risk study, we dropped the requirement for informed consent.

### 2.2 Therapeutic methods

Combined therapy was performed by three qualified radiologists and hepatologists with more than 5 years of experience. The femoral artery was punctured using a modified Seldinger method, in which a microcatheter was inserted into the supplying artery of the tumor and doxorubicin (Pfizer, USA) and lipiodol (Gabcod, France) were injected. The interruption of blood flow in the tumor supplying artery was considered complete embolization.Local ablation was performed within 2 weeks after TACE. After the patient has underwent local anesthesia,the procedure is performed percutaneously by hepatologists under the guidance of triphasic computed tomography (CT) or magnetic resonance imaging (MRI). According to the number and size of tumor, overlapping ablation, multi-site ablation and fractional ablation were performed respectively. To ensure complete ablation, a safe distance of 0.5-1.0cm should be maintained around the tumor.

In order to ensure complete ablation of large tumors and multiple tumors, we use the combined treatment of TACE plus ablation, first reducing the target lesion by TACE to facilitate the subsequent complete ablation.On the one hand, TACE can block the blood supply and mark the tumor; on the other hand, TACE is beneficial for subsequent ablation; ablation therapy can further inactivate residual lesions, and ablation treatment is a minimally invasive, efficient and reproducible treatment, so for patients difficult to complete primary ablation, repeated ablation is used to achieve complete ablation.

### 2.3 Follow-up

Patients follow-up were performed on outpatient clinic. Follow-up consisted of physical examination, blood tests, and imaging examination, which included abdominal ultrasound every 3-6 months and ontrast-enhanced CT/MRI every 6 months. The follow-up involved a physical examination and blood tests, as well as imaging, included an abdominal ultrasound every 3–6 months and contrast-enhanced CT/MRI every 6 months. Recurrence was defined as intrahepatic local progression, distal intrahepatic recurrence and extrahepatic metastases. RFS was calculated as the time between the date of ablation and the patient’s first evidence-based recurrence or death in patients without evidence of disease recurrence, whereas OS was defined as the time between the date of ablation and tumor-related death or the date of the last visit. The cut-off date for this study is July 1, 2020. Patients were treated with radiofrequency ablation or TACE when they were found to have tumor recurrence. Contrast-enhanced CT and/or MRI is used to determine if a patient has recurrence. Recurrence is considered when a patient’s imaging shows enhancement of areas within or around the primary tumor.

### 2.4 Statistical analysis

Data were analyzed using SPSS 26.0 software (IBM, Armonk, NY, USA), and all figures were created by SPSS and Graphpad Prism 8.0(Graphpad software Inc). Continuous variables were expressed as the mean ± standard deviation (SD), and categorical data were presented as the frequency. Univariate and multivariate Cox regression analyses were performed to assess the independent risk factors of prognosis in HCC patients undergoing combined therapy. The RFS and OS rates were calculated with the Kaplan-Meier method, and the differences between groups were compared by using the Log-rank test. Statistical significance was considered when the P value < was 0.05.

## 3 Results

### 3.1 Baseline characteristics and follow-up results

The present study consisted of 90 men (81.1%) and 21 women (18.9%) with a mean age of 56 years ± 9 years. Additionally, 27 patients (24.3%) had hypertension, 55 patients (49.5%) were received antiviral therapy before they underwent combined therapy. There were 50 patients (45.0%) who had a history of smoking and 33 patients (29.7%) with a history of drinking. By the end of follow-up, 88 (79.3%) recurred and 43 (38.7%) died. The median follow-up duration was 52.2 (36.4-64.9) months. The 1-,3- and 5-year RFS rates were 60.4% (67/111), 29.7% (33/111),and 20.7%(23/111). Moreover, the 1-, 3-, and 5-year cumulative OS rates were 100%(111/111), 73.0% (81/111) and 61.3% (68/111), respectively. There were differences in age (P=0.040) and lymphocyte count (P=0.015) between the recurrence group and the non-recurrence group (**Table 1**).

**Table 1:** Comparison of data between recurrent and non-recurrent HCC patients.

Variables	Total	Non-Recurrence	Recurrence	P
Gender, male/female	90/21	20/4	70/17	0.981
Age,≤60 years/>60 years	73/38	20/4	53/34	**0.040**
Hypertension,yes/no	27/84	9/15	18/69	0.089
Antiviral,yes/no	51/60	11/13	40/47	0.990
Smoking,yes/no	50/61	11/13	39/48	0.930
Drinking,yes/no	33/78	6/18	27/60	0.567
Family,yes/no	54/57	13/11	41/46	0.541
Cirrhosis,yes/no	86/25	20/4	66/21	0.438
Tumor size,≤3cm vs>3cm	57/51	12/12	45/39	0.757
Tumor number, solitary/multiple	78/26	19/5	59/21	0.591
AFP,≤7ng/mL vs>7ng/mL	49/62	9/15	40/47	0.459
Ki67,≤10% vs>10%	63/48	16/8	47/40	0.268
GPC3,Negative/Positive	14/96	0/24	14/72	0.077
P53,Negative/Positive	24/84	6/18	18/67	0.690
Tumor differentiation,poor/middle/well	30/60/20	8/14/2	22/46/18	0.344
HBsAg,Negative/Positive (%)	46/53	12/9	34/44	0.269
ALT (U/L)	45.83 ± 29.43	47.46 ± 33.59	45.14 ± 27.62	0.639
AST (U/L)	35.17 ± 17.75	37.85 ± 22.77	34.43 ± 16.19	0.406
Total serum bilirubin (μmol/L)	17.16 ± 8.41	14.75 ± 6.78	17.82 ± 8.73	0.073
ALB (g/L)	37.76 ± 4.48	38.74 ± 3.78	37.48 ± 4.64	0.225
γ-GT (u/L)	62.20 (38.90,86.20)	64.00 (44.75,81.05)	61.50 (36.20,87.70)	0.788
HBsAg (serum),Negative/Positive	1/106	0/23	1/83	0.785
PLT (10^9/L)	137.25 ± 59.04	124.71 ± 51.79	140.74 ± 60.73	0.241
LYM (10^9/L)	1.29 ± 0.58	1.09 ± 0.39	1.35 ± 0.61	**0.015**
NEU (10^9/L)	4.01 ± 1.89	3.96 ± 1.84	4.02 ± 1.92	0.890
PT	11.94 ± 1.06	11.70 ± 0.77	12.00 ± 1.12	0.134
PTA	92.12 ± 12.67	94.08 ± 9.05	91.57 ± 13.49	0.290
Fib (g/L)	3.13 ± 0.99	2.99 ± 0.91	3.16 ± 1.01	0.456

Bolded values indicates P<0.05, which is statistically significant.

### 3.2 Indicators that correlate with RFS

Correlations between demographic and pathological indicators and clinical laboratory data and RFS were assessed with univariate and multifactorial analyses. The univariate analysis demonstrated that RFS was significantly associated with age, intrahepatic HBsAg, albumin, total serum bilirubin, PT and PTA. The multivariate analysis showed that intrahepatic HBsAg expression (HR: 1.965; 95%CI: 1.169-3.304) was an independent predictor of HCC recurrence (P<0.05) (**Table 2**)

**Table 2:** Prognostic factors for RFS by Cox proportional hazards regression model.

Variables	Univariate		Multivariate	
	HR (95%)	P value	HR (95%)	P value
Gender	0.983 (0.578-1.673)	0.949		
Age	1.567 (1.012-2.428)	**0.044**	1.161 (0.690-1.954)	0.574
Hypertension	0.731 (0.435-1.228)	0.236		
Antiviral	1.006 (0.699-1.627)	0.766		
Smoking	1.022 (0.668-1.564)	0.919		
Drinking	0.997 (0.631-1.574)	0.989		
Family	0.908 (0.596-1.385)	0.654		
Cirrhosis	1.060 (0.647-1.737)	0.817		
Tumor size	1.039 (0.676-1.597)	0.86		
Tumor number	1.350 (0.815-2.235)	0.244		
AFP	0.933 (0.611-1.424)	0.747		
Ki67	1.128 (0.739-1.722)	0.578		
GPC3	0.698 (0.391-1.246)	0.224		
P53	0.936 (0.556-1.577)	0.804		
Tumor differentiation	1.253 (0.908-1.728)	0.17		
HBsAg	1.716 (1.090-2.701)	**0.02**	1.965 (1.169-3.304)	**0.011**
ALT (U/L)	0.999 (0.992-1.006)	0.781		
AST (U/L)	0.997 (0.985-1.008)	0.572		
Total serum bilirubin (μmol/L)	1.024 (0.999-1.049)	**0.06**	1.028 (0.997-1.061)	0.081
ALB (g/L)	0.956 (0.911-1.005)	**0.075**	1.012 (0.951-1.077)	0.714
γ-GT (u/L)	1.004 (1.001-1.008)	**0.015**	1.004 (0.999-1.008)	0.100
PLT (10^9/L)	1.000 (0.997-1.004)	0.871		
LYM (10^9/L)	1.243 (0.859-1.798)	0.249		
NEU (10^9/L)	0.977 (0.875-1.091)	0.684		
HBsAg (serum)	0.772 (0.107-5.582)	0.798		
PT	1.281 (1.041-1.576)	**0.019**	2.294 (0.929-5.665)	0.072
PTA	0.983 (0.966-1.001)	**0.059**	1.060 (0.985-1.140)	0.122
Fib (g/L)	1.134 (0.919-1.400)	0.242		

Bolded values indicates P<0.05, which is statistically significant.

### 3.3 Indicators that correlate with OS

Univariate and multivariate analyses were performed to assess the relationship between demographic, pathological indicators and clinical laboratory data and OS. Univariate analysis showed that OS was significantly correlated with cirrhosis, antiviral, intrahepatic HBsAg expression, GGT, PT and PTA. The multivariate analysis showed that intrahepatic HBsAg expression (HR:2.320;95%CI:1.093~4.925)and antiviral(HR:3.272;95%CI:1.480~7.234) were an independent predictor of HCC survival status (P<0.05) (**Table 3**).

**Table 3:** Prognostic factors for OS by Cox proportional hazards regression model.

Variables	Univariate		Multivariate	
	HR (95%)	P value	HR (95%)	P value
Gender	0.815 (0.361-1.840)	0.622		
Age	1.423 (0.757-2.676)	0.273		
Hypertension	0.813 (0.374-1.767)	0.601		
Antiviral	1.739 (0.933-3.244)	**0.082**	3.272 (1.480-7.234)	**0.003**
Smoking	1.484 (0.802-2.743)	0.209		
Drinking	1.442 (0.762-2.728)	0.26		
Family	0.870 (0.470-1.609)	0.657		
Cirrhosis	2.443 (0.958-6.232)	**0.062**	1.193 (0.433-3.290)	0.733
Tumor size	0.947 (0.502-1.786)	0.866		
Tumor number	1.187 (0.578-2.440)	0.64		
AFP	1.277 (0.681-2.393)	0.446		
Ki67	1.027 (0.508-1.756)	0.858		
GPC3	0.660 (0.292-1.489)	0.316		
Tumor differentiation	1.172 (0.738-1.862)	0.501		
HBsAg	2.625 (1.254-5.496)	**0.01**	2.320 (1.093-4.925)	**0.028**
ALT (U/L)	0.998 (0.987-1.008)	0.661		
AST (U/L)	1.000 (0.984-1.016)	0.983		
Total serum bilirubin (μmol/L)	1.001 (0.966-1.038)	0.937		
ALB (g/L)	0.931 (0.870-0.996)	**0.039**	0.997 (0.908-1.094)	0.945
γ-GT (u/L)	1.005 (1.001-1.009)	**0.009**	1.004 (1.000-1.009)	0.062
PLT (10^9/L)	0.998 (0.993-1.004)	0.55		
LYM (10^9/L)	1.101 (0.625-1.941)	0.738		
NEU (10^9/L)	0.935 (0.789-1.108)	0.439		
HBsAg (serum)	0.414 (0.057-3.032)	0.385		
PT	1.463 (1.099-1.946)	**0.009**	1.790 (0.461-6.960)	0.400
PTA	0.972 (0.947-0.996)	**0.024**	1.020 (0.914-1.138)	0.728
Fib (g/L)	0.950 (0.694-1.300)	0.747		

Bolded values indicates P<0.05, which is statistically significant.

### 3.4 Analysis of clinical and prognostic data based on intrahepatic HBsAg expression

**Figure 2:**
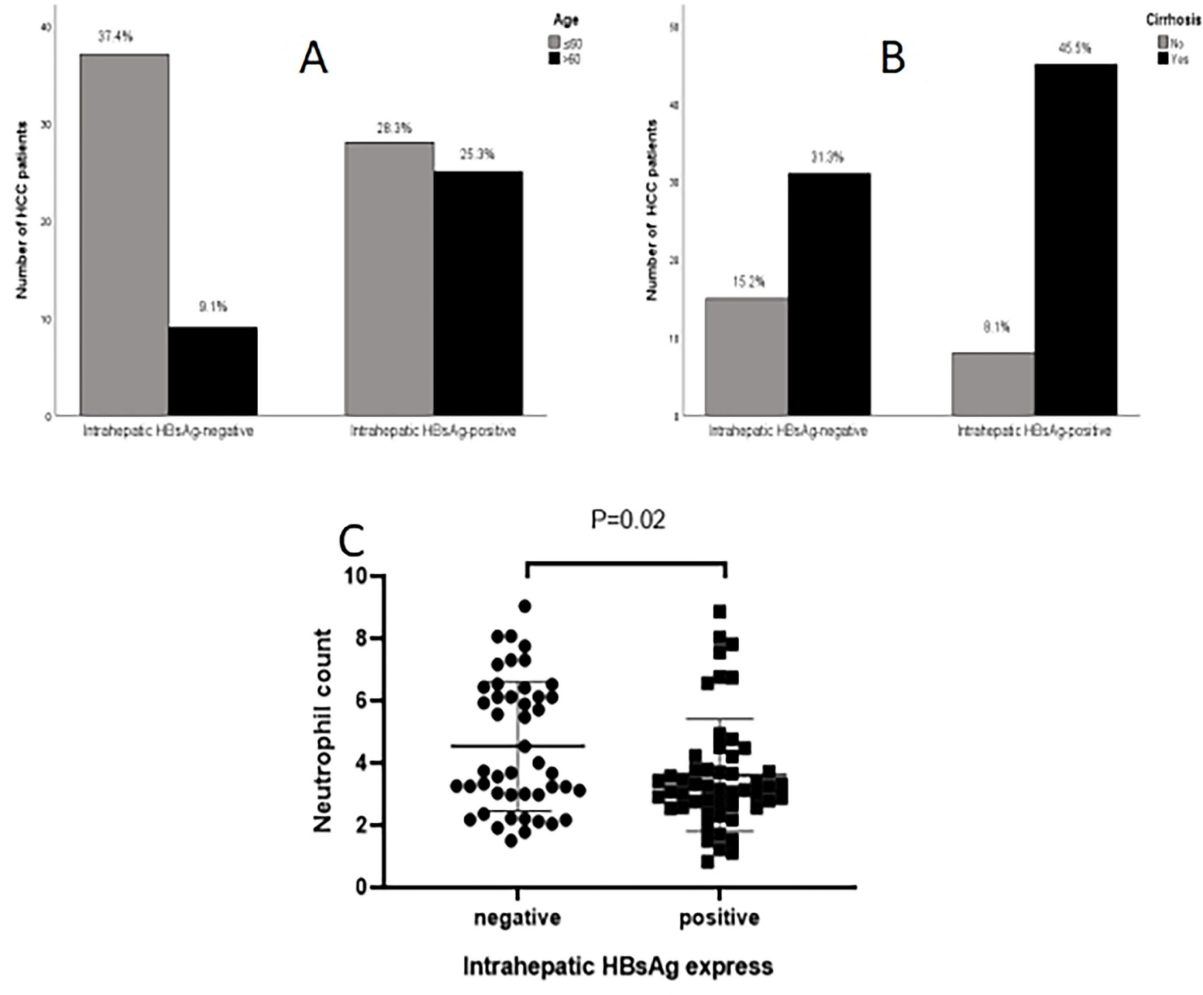
Comparison of clinical data based on intrahepatic HBsAg. **(A)** Age; **(B)** Cirrhosis; **(C, D)** neutrophil count.

To investigate the influence of intrahepatic HBsAg expression on patient prognosis, patients were divided into two groups according to the expression of intrahepatic HBsAg; one group was positive intrahepatic HBsAg expression, and the other one was negative intrahepatic HBsAg expression. Statistical analysis showed that the age (P=0.004), cirrhosis (P=0.040) and neutrophil count (P=0.020) were significant difference between the two group (**Table 4**; **Figure 2**).

**Table 4:** Comparison of clinical data based on intrahepatic HBsAg.

Variables	HBsAg		P
	Negative	Positive	
Gender, male/female (%)	37/9	42/11	0.883
Age,≤60 years/>60 years (%)	37/9	28/25	**0.004**
Hypertension,yes/no (%)	13/33	10/43	0.270
Antiviral,yes/no (%)	20/26	27/26	0.458
Smoking,yes/no (%)	19/27	22/31	0.984
Drinking,yes/no (%)	16/30	14/39	0.366
Family,yes/no (%)	21/25	27/26	0.599
Cirrhosis,yes/no (%)	31/15	45/8	**0.040**
Tumor size,≤3cm vs>3cm (%)	23/23	31/20	0.286
Tumor number, solitary/multiple (%)	35/10	34/14	0.444
AFP,≤7ng/mL vs>7ng/mL (%)	23/23	24/29	0.639
Ki67,≤10% vs>10% (%)	27/19	28/25	0.588
GPC3,Negative/Positive (%)	4/41	10/43	0.159
P53,Negative/Positive (%)	11/35	11/40	0.783
Tumor differentiation,poor/middle/well (%)	15/24/7	13/28/12	0.528
ALT (U/L)	45.64 ± 32.76	44.00 ± 25.92	0.782
AST (U/L)	37.60 ± 23.16	33.78 ± 13.07	0.326
Total serum bilirubin (μmol/L)	18.67 ± 9.01	15.97 ± 7.32	0.108
ALB (g/L)	38.72 ± 4.38	37.16 ± 4.44	0.083
γ-GT (u/L)	61.95 (38.98-83.53)	54.30 (35.85-84.45)	0.700
PLT (10^9/L)	137.32 ± 57.37	135.67 ± 61.13	0.891
LYM (10^9/L)	1.32 ± 0.54	1.28 ± 0.63	0.703
NEU (10^9/L)	4.54 ± 2.08	3.60 ± 1.80	**0.020**
HBsAg (serum)	1/44	0/51	0.469
PT	11.82 ± 1.10	11.94 ± 0.97	0.545
PTA	94.00 ± 13.10	91.85 ± 11.95	0.395
Fib (g/L)	3.16 ± 1.06	3.07 ± 0.90	0.663

Bolded values indicates P<0.05, which is statistically significant.

**Figure 3:**
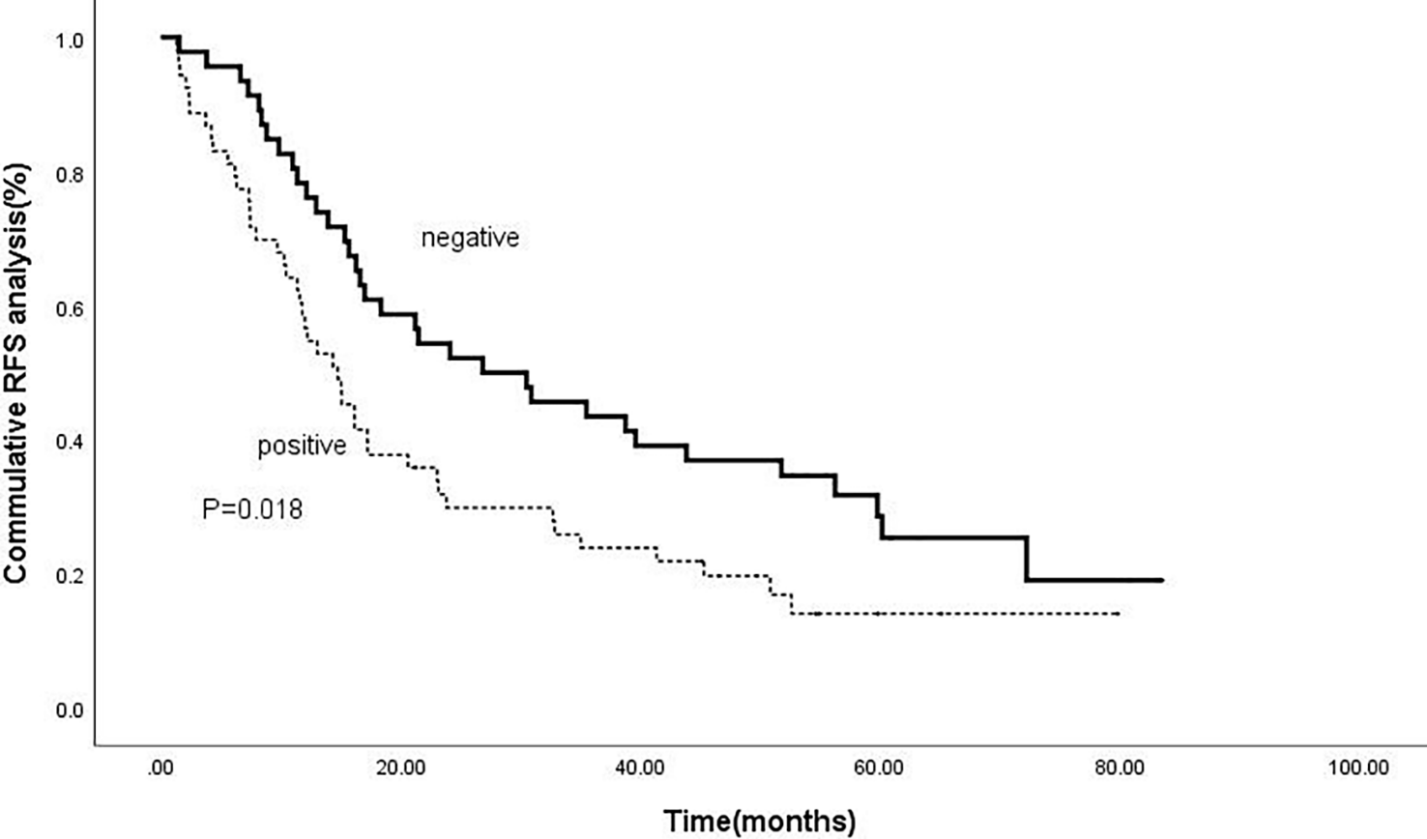
Comparison of RFS based on intrahepatic HBsAg. The cumulative 1-, 3-, and 5-year RFS rates for patients with negative HBsAg expression after combined therapy were 78.3%, 43.5% and 30.4%; while for patients with positive HBsAg expression were 58.5%, 24.5% and 17.0%, respectively (P=0.018).

**Figure 4:**
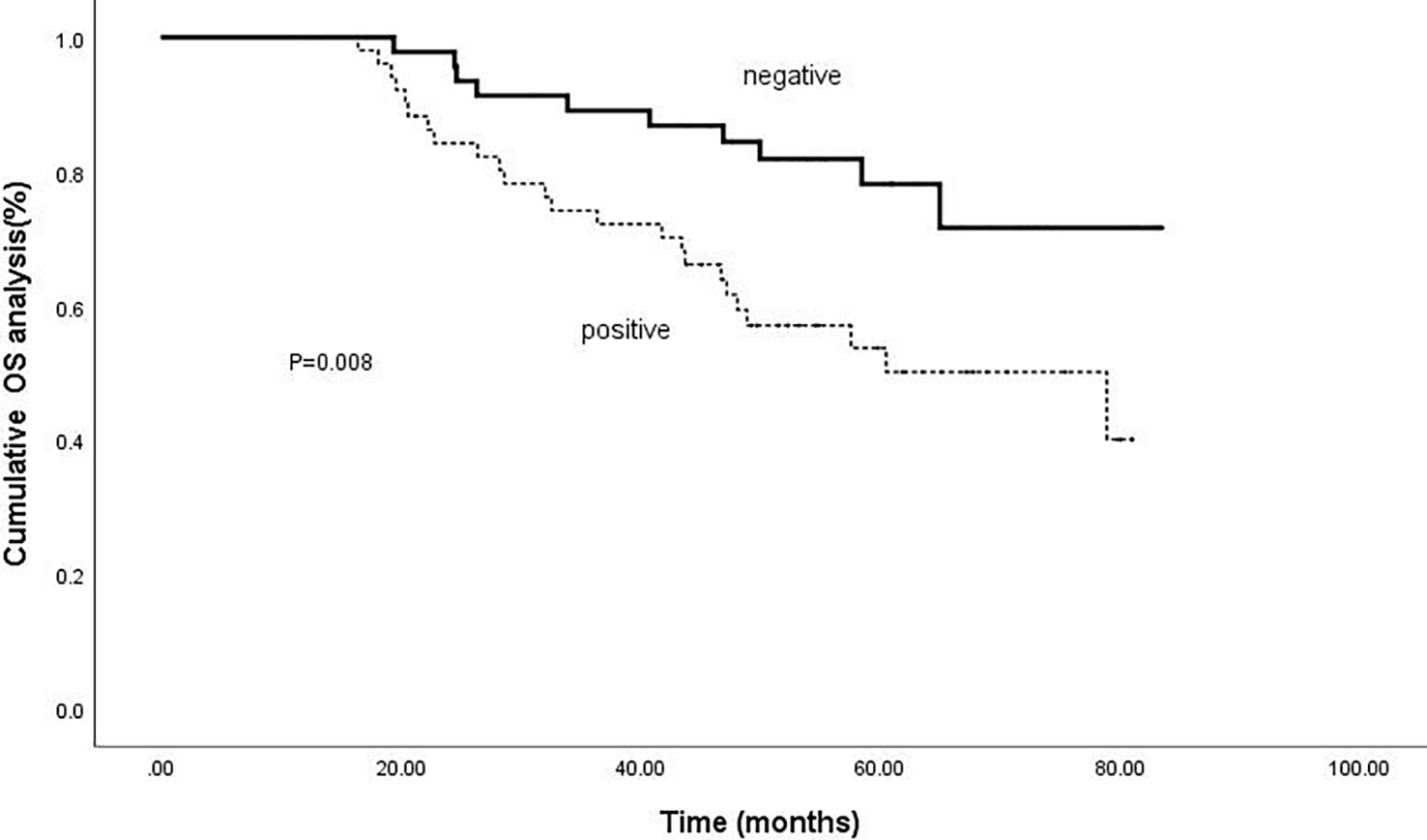
Comparison of OS based on intrahepatic HBsAg. The cumulative 1-, 3-, and 5-year OS rates for patients with negative HBsAg expression after combined therapy the were 100%, 89.1% and 80.4%; for patients with positive HBsAg expression were 100%, 75.5% and 58.5%, respectively (P=0.008).

Kaplan-meier analysis confirmed that positive intrahepatic HBsAg expression was a negative predictor of RFS and OS. The cumulative 1-, 3-, and 5-year RFS rates for patients with negative HBsAg expression after combined therapy were 78.3%, 43.5% and 30.4%; while for patients with positive HBsAg expression were 58.5%, 24.5% and 17.0%, respectively (P=0.018).(**Figure 3**) Meanwhile, the cumulative 1-, 3-, and 5-year OS rates for patients with negative HBsAg expression after combined therapy the were 100%, 89.1% and 80.4%, while, for patients with positive HBsAg expression were 100%, 75.5% and 58.5%, respectively (P=0.008) (**Figure 4**).

## 4 Discussion

The incidence of HCC is very high and increasing in developing countries, especially in China. And its incidence and mortality rates continue to increase (8). It has been reported that even in patients with early-stage (BCLC-0/A), the 5-year recurrence rate is 50-85% (9–13). As a result, HCC has increased the burden of medical care in our country and become a serious health problem (14).

The results of this study suggest that antiviral treatment was an independent risk factor for OS. A number of previously conducted studies indicated that HBV reactivation is the main risk factor for liver cancer recurrence (15–17), postoperative antiviral therapy can reduce viral load and liver inflammation, improve liver function and the prognosis of patients, and reduce HCC recurrence and mortality. The current research showed that antiviral treatment can effectively improve the outcomes of patients who received surgical and ablation therapy while improve the survival rate of patients (18).

HBV infection to the progression of cancer is a multi-step process (19), HBsAg promotes the proliferation of HCC cells through the activation of the Src/pi3k/Akt pathway (20). Chronic inflammation caused by chronic viral infection leads to changes in the liver microenvironment and increases increase the risk of HCC development. The results of this study confirm that intrahepatic HBsAg positive expression is an independent risk factor for predicting OS and RFS in patients who received combined therapy. The study also showed that the OS and RFS of negative express patients were better than those with positive HBsAg expression.

The results of this study showed there was no correlation between HBsAg in serum and HBsAg in liver tissues. This is consistent with the results of the studies reported in previous articles (21). However, the results of the present study indicated that serum HBsAg expression is associated with intrahepatic HBsAg expression, the paper also suggested that not all serum HBsAg expression was consistent with intrahepatic HBsAg expression (22), the negative correlations observed between low liver HBsAg levels and the increased expression of T- and B cell-activated genes may point toward a limited HBsAg-induced immune exhaustion and enhanced immune control and/or a higher proportion of leukocytes in the livers of these patients. Therefore, for patients with positive intrahepatic HBsAg expression, follow-up strategies should be enhanced to monitor tumor progression more closely and to help physicians take timely interventions to decrease the recurrence rate and improve the long-term prognosis of patients.

The prognosis of HCC patients is still poor. Thus, it is crucial to explore the biological indicators that can predict patients’ prognosis and make the corresponding clinical decisions according to the patients’ situation. In addition, this study is a single-center study while also a small sample study, thus required more centers and a larger sample sizes participation in the validation.

## 5 Conclusions

Intrahepatic HBsAg positive expression is associated with poor prognosis of HCC patients who underwent combined therapy. Accordingly, for patients with positive HBsAg express in liver tissue, corresponding clinical decisions should be made to improve patients the long-term prognosis of patients.

## Data availability statement

The data analyzed in this study is subject to the following licenses/restrictions: The data used to support the findings are available from the corresponding author upon request. Requests to access these datasets should be directed to zhangyh@ccmu.edu.cn.

## Ethics statement

The study has been approved by the ethics committee of the Beijing You’an Hospital affiliated to Capital Medical University. As a minimum risk study that was in accordance with the Helsinki protocol, the requirement for patients’ informed consent was waived by the same ethics committee that approved the study (Beijing You’an Hospital affiliated to Capital Medical University), and all methods were carried out in accordance with relevant guidelines and regulations. Written informed consent for participation was not required for this study in accordance with the national legislation and the institutional requirements.

## Author contributions

YZ conceived and designed the protocol. JZ and YZ collected the data. BL and QW wrote the manuscript. TM and KL analyzed the data. WG and CY critically revised the manuscript. All authors contributed to the article and approved the submitted version.
